# Improving the Biological Properties of UHMWPE Biocomposite for Orthopedic Applications

**DOI:** 10.1155/2023/4219841

**Published:** 2023-01-12

**Authors:** Tamara R. Kadhim, Jawad K. Oleiwi, Qahtan A. Hamad

**Affiliations:** Materials Engineering Department, University of Technology, Baghdad, Iraq

## Abstract

Bone plates are essential for bone fracture healing because they modify the biomechanical microenvironment at the fracture site to provide the necessary mechanical fixation for fracture fragments. The objective of this study was to determine cell availability, antibacterial activity, and wettability through a contact angle test. However, biocomposites that involve UHMWPE reinforced with n-HA and n-TiO_2_ particles at different fractions (0, 1.5, 2.5, 3.5, and 4.5%) and 5% from carbon and Kevlar fibers were fabricated by hot pressing technique. In vitro studies revealed good cell viability on the surface of the hybrid biocomposite even after 72 hr. The UHMEPE nanocomposite reinforced with carbon showed better cell attachment for fibroblasts than other UHMWPE nanocomposite materials reinforced with Kevlar fiber. The results of the contact angle measurements indicated that the incorporation of nanoparticles and the fiber reinforcement increased the wettability due to the hydrophilic character of nanobiocomposite, and also (UHMWPE-4.5% wt. TiO_2_–CF) biocomposite was the best wettability (∼48% as compared to neat UHMWPE). Antibacterial experiments involving Gram-positive bacteria, *Staphylococcus aureus,* confirm excellent bactericidal property for (UHMWPE-4.5% wt. TiO_2_–CF) biocomposite. Thermal analysis of the produced nanocomposites revealed that they had higher melting and crystallinity temperatures than pure UHMWPE.

## 1. Introduction

When a human bone fracture occurs, various types of internal fixation devices, such as bone plates, are placed at the fracture site to help stabilize the bone structure [1]. Metals such as stainless steel, titanium, and their alloys are not the best material for a bone plate because of the negative effects on callus formation and fracture healing caused by the high modulus of elasticity and biomechanical mismatch to the bone [2, 3]. Polymer-based composite, which has reduced stiffness for bone plate fixations, can be used as an alternative to metal materials to solve these problems. Due to its high chemical resistance, biocompatibility, and mechanical and tribological properties, ultrahigh molecular weight polyethylene (UHMWPE) is a polymer that is frequently employed in medical applications. The biological internal fixation employing the internal fixator principle exhibits an indirect healing pattern and a low infection rate, which was firmly established with a very high follow-up rate of 97% [4]. Bonfield et al. introduced hydroxyapatite (HA)-reinforced high-density polyethylene (HDPE), and there has been a continuous effort to develop bone-analogue composites for biomedical applications. However, these composites' lower strength and stiffness to the cortical bone have restricted their use as load-bearing bone replacements [5]. In a recent study, scientists created new plate fixation using thermoplastic composite polymer. Due to their biocompatibility and degradation rates, which are readily modified by changing the composition and production method, thermoplastic polymers are more advantageous than thermoset polymers [6, 7]. According to previous researchers, second phases of ceramics (A1_2_O_3_, TiO_2_, quartz, wollastonite, kaolin, CaCO_3_, etc.), carbons (carbon black, carbon fiber, carbon nanotubes, graphite), and polymers (polyurethane, phenyl p-hydroxyzoate, etc.) can improve the mechanical properties of UHMWPE composites [8–10]. Hydroxyapatite (HA, Ca_10_(PO_4_)_6_(OH)_2_), the main inorganic component of hard tissues, has a variety of applications in bone fillers and replacements due to its excellent bioactivity and osteoconductivity [11]. Celebi Efe et al. investigated whether UHMWPE–TiO_2_ composite films meet basic requirements for biological applications of artificial hip joint acetabular liner materials [12]. In this research analysis, wettability through contact angle measurement enhanced the antibacterial activity of n-HA and n-TiO_2_ biocomposite and CF and KF hybrid biocomposite plates fixation and studied the cytotoxicity of UHMWPE biocomposites with human fibroblast cells.

## 2. Materials and Methods

**Figure 1:**
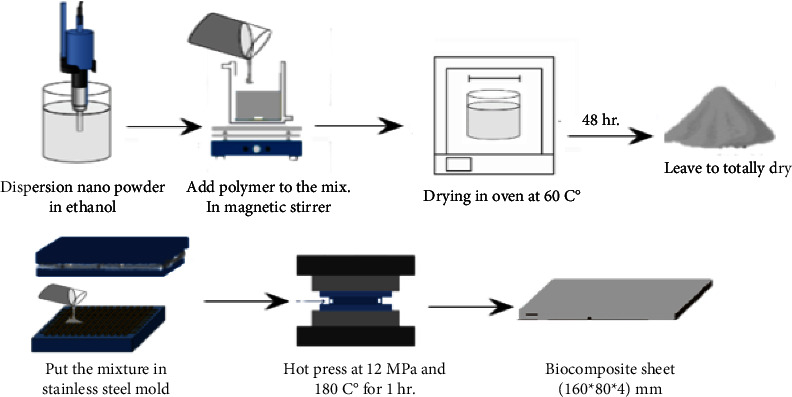
Procedure of manufactured biocomposite materials as bone plate fixation.

UHMWPE polymer powder with molecular weight 600–700 (104 g/mol.) and density (0.093–0.94) (g/cm^3^) was supplied by the Luoyang Max Pipe Industry as a matrix. Hydroxyapatite nanopowder and titanium oxide (anatase phase) nanopowder were obtained from Xian Real and Hangzhou Union in Biotechnology Company, China, as reinforcement materials. The materials were weighed by weight fraction (0, 1.5, 2.5, 3.5, and 4.5%). Firstly, the powder particles are dispersed in ethanol with an ultrasonic device for 45 min for n-HA and 30 min for n-TiO_2_. Secondly, the UHMWPE is added to the nanoparticles simultaneously, followed by mechanical mixing for 30 min to n-HA and 15 min to n-TiO_2_ at 1500 rpm. To violate the ethanol, we then simply place the mixture in an oven at 60°C for 2 hours and allowed it to stand for 48 hours, tightly dry. Thereafter, the mixture was placed in a mold and pressed in a hydraulic press at a temperature of 180°C and a pressure of 12 MPa for one hour. Then, the mold left to cool in air up to room temperature to get the sheet of composite material as shown in Figure 1. After achieved all tests on the prepared particulate biocomposite materials, it is found that the best composite materials are (UHMWPE+4.5% n-HA) and (UHMWPE+4.5% n-TiO2) and it is chosen to fabricate the hybrid biocomposites by the addition of two types of fibers (Kevlar and carbon) as one layer. Then, the hot press was achieved by the same procedures which were previously mentioned for the preparation of particulate biocomposite material.

### 2.1. Antibacterial Activities

The antibacterial potential of the prepared samples (1, 2, 3, 4, 5, 6, 7) was investigated against Gram's negative and Gram's positive bacterial strains using an agar well diffusion assay [13, 14]. About 20 mL of Muller–Hinton (MH) agar was aseptically poured into sterile Petri dishes. Muller–Hinton (MH) agar was aseptically placed in the amount of 20 mL onto sterile Petri plates. From their stock cultures, the bacterial species were separated using a sterile wire loop [14]. After the organisms had been cultivated, 6-mm-diameter wells on the agar plates were bored using sterile points. The samples (1, 2, 3, 4, 5, 6, 7) were injected into the bored wells at a variety of concentrations. Prior to calculating and recording the average zones of inhibition diameter, the cultivated plates containing the samples (1, 2, 3, 4, 5, 6, 7) and the test organisms were incubated overnight at 37°C [15, 16].

### 2.2. Contact Angle (Wettability Test)

Many biological, chemical, and physical processes depend on the wettability of a surface. The contact angle, which is the angle created by the tangent to the liquid-vapor interface and the solid surface at the three-phase contact line, is frequently used to describe wetting [17]. The contact angle was measured according to the ASTM stander (D5946-04) using optical contact angle and interface tension meter [18]. The specimen was placed on a glass slide; the tissues were then inserted into the instrument specimen holder after being tightened for better observation during the contact angle measurements. Then, a distilled water droplet with a volume of 8 ml was dropped onto the biocomposite surface. After dipping, the contact angle measurement was taken, and a video camera captured the droplet shape.

### 2.3. MMT Assay (Cell Availability)

In order to quantify the cell growth, 100 L of DMEM/F12 supplemented with 10% heat-inactivated fetal bovine serum (FBS) was added to the cells before they were seeded in a 96-well tissue culture plate at a density of 104 cells per well. This 24-hour incubation period was involved. The cells were then treated for 4 hours with a fresh, serum-free culture medium that contained serial dilutions of the sample. Then, for a second 24 hours, the media were changed with 100 L of brand-new full media. Cells were then incubated for a further 4 hours at 37°C after the medium was replaced with 100 L of fresh medium containing MTT, giving a final MTT concentration of 0.5 mg/ml. The medium was aspirated after 4 hours, and each well's absorbance (570 nm) was measured using a microplate reader after the MTT formazan produced by living cells was dissolved in 100 L of DMSO. The absorbance values of the sample-treated wells and control wells (the control cells grown in a medium without the CDs), respectively, were used to calculate the relative cell viability (%). Data are provided as average SD (*n* = 3) [19].

## 3. Result and Discussions

### 3.1. Characterization

**Figure 2:**
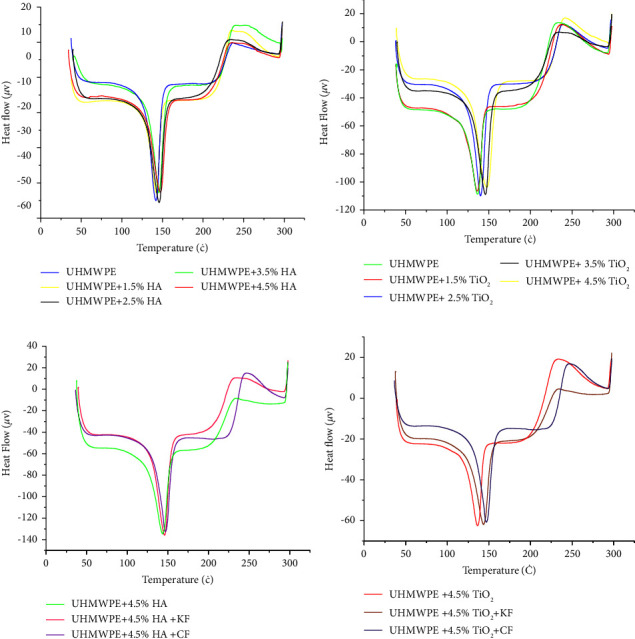
DSC analysis for (a) particulate biocomposite with hydroxyapatite NPs, (b) particulate biocomposite with titanium oxide NPs, (c) hybrid biocomposite with hydroxyapatite NPs, and (d) hybrid biocomposite with titanium oxide NPs.

The purpose of this test was to evaluate the thermal behavior and physical changes that occurred when pure UHMWPE and UHMWPE biocomposite specimens were heated. Differential scanning calorimetry (DSC) measurements were carried out according to ASTM D3418-03 under a nitrogen gas atmosphere. The prepared samples with weight of (8–10) ± 0.5 mg were mounted in aluminum pans and heats up from −40 to 250°C with a heating rate of 10°C/min [20]. Thermal properties of biocomposite materials (UHMWPE + n-HA wt. %) and (UHMWPE + n-TiO_2_ wt. %) with variation weight fraction of NPs using the DSC inspection are illustrated in Figure 2. The melting and crystallization temperatures of polymer composites were measured using differential scanning calorimetry (DSC), as shown by the curves. Percentages are summarized in Table 1.

From the DSC analysis represented in Figure 2, it is clear that the melting point of UHMWPE increased from 136.29°C for neat UHMWPE polymer to 145 and 144.82 for n-HA and, n-TiO_2_, respectively, for weight fraction of 4.5%. It can be noticed that the melting temperature increase with the increase (HA and TiO_2_) in nanoparticles but the higher values was obtained in (UHMWPE/n-HA) biocomposite compared to (UHMWPE/n-TiO_2_) biocomposite due to better compatibility [10, 21]. This indicates that the 4.5% wt. of NPs has the strongest nucleation effect that promotes the formation of microcrystalline zones within the biocomposite [22, 23]. However, the effect of fiber reinforcement on the melting points of samples was more remarkable and reaches 148.27°C at (UHMWPE + 4.5% HA + CF) due to the uniform distribution of the fiber within the matrix and also to their thermal properties of the carbon fibers [24]. Moreover, crystallization temperature was increasing when the addition of NPs. The higher thermal stability of the biocomposites as compared with the neat UHMWPE is attributed to the formation of a cross-linked network upon chain scission and improved compactness of the polyethylene.

### 3.2. Contact Angle

**Figure 3:**
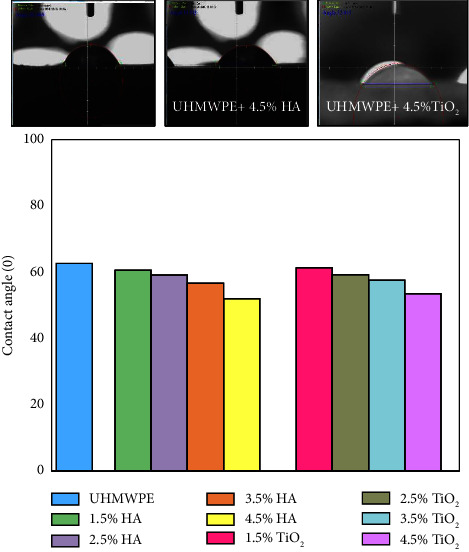
Contact angle for particulate biocomposites as a function of HA and TiO_2_ NPs.

Figure 3 illustrates the contact angle of (n-HA) and (n-TiO_2_) particulate biocomposites. It can be seen from the figure that the incorporation of HA and TiO_2_ NPs caused the contact angle to decrease with an increase in wt.% of nanoparticles, from (62.66) for pure UHMWPE to (53.54), (52.04) for 4.5% n-HA and 4.5% n-TiO_2_, respectively. This may be explained by the fact that the presence of nanoparticles in excess of a threshold quantity may cause HA and TiO_2_ deposited on the surface of nanobiocomposites to reduce surface roughness, making the surfaces more compact and reducing their hydrophobicity [25, 26].

**Figure 4:**
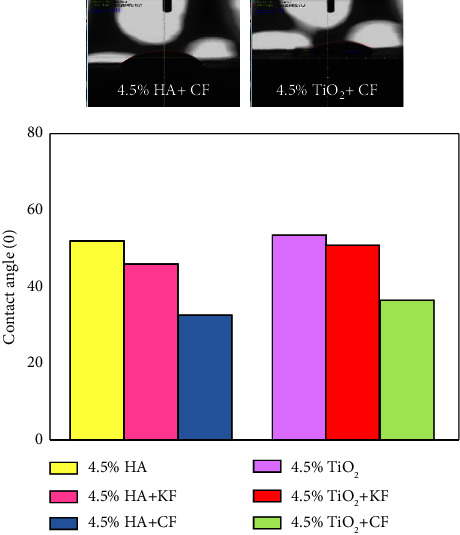
Contact angle for hybrid biocomposites as a function of types of fibers.

Figure 4 illustrates the contact angle of hybrid biocomposites, and the wetting behavior for Kevlar and carbon fiber represents a high reduction in contact angle and has a hydrophilic character compared to 4.5% n-HA and 4.5% n-TiO_2_ particulate biocomposites. Carbon fiber when added to particulate biocomposites shows excellent wettability as compared to Kevlar fiber which has (32.57) for (4.5% n-HA + CF) whenever (45.96) for (4.5% n-HA + KF). The hydrophilicity is a direct relation to biocompatibility; the smallest the contact angle the better the biocompatibility [27].

### 3.3. Antibacterial Activity

**Figure 5:**
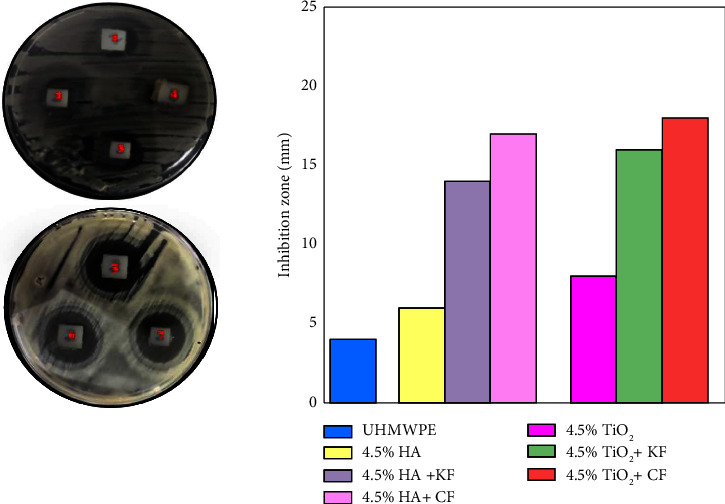
Inhibition zone of the n-HA and n-TiO_2_ particulate and hybrid biocomposites against *S. aureus* bacteria.

**Figure 6:**
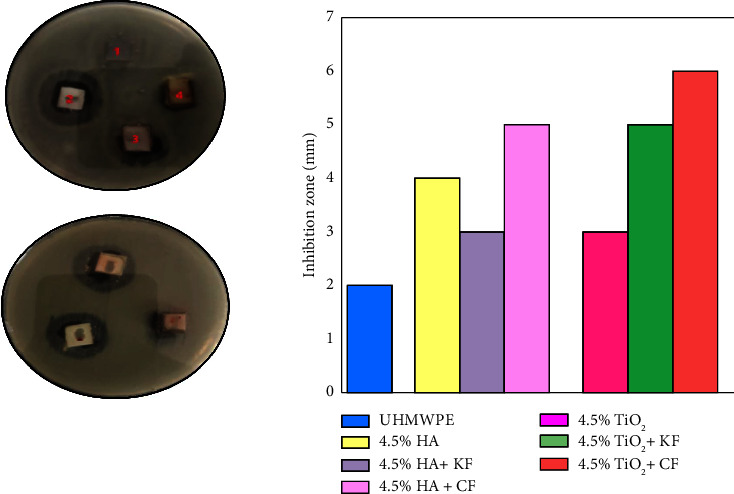
Inhibition zone of the n-HA and n-TiO_2_ particulate and hybrid biocomposites against *E. coli* bacteria.

The antibacterial activity of the samples is evaluated against two bacterial types *S. aureus* and *E. coli*. Figures 5 and 6 presented the inhibition zone for biocomposites samples against *E. coli* and *S. aureus* bacteria. The positions (1–7) at the top correspond to UHMWPE, HA, HA + KF, and HA + CF and at the bottom TiO_2_, TiO_2_ + KF, and TiO_2_ + CF biocomposites, respectively. The highest inhibition zone values are obtained by n-TiO_2_ biocomposite. It was explained that n-TiO_2_ shows its antibacterial activity. The anatase crystalline structure of TiO_2_ presents its highest antibacterial activity among other crystalline structures which is an important condition that affects its physicochemical properties, which in turn affects its antibacterial activity [28, 29]. Depending on the result, it can be noticed that the antibacterial efficiency of UHMWPE biocomposites to the *S. aureus* bacteria is more than that of antibacterial efficiency to *E. coli* bacteria [30]. Furthermore, when particulate biocomposite was combined with fibers, it was shown that (UHMWPE+n-TiO_2_+CF) hybrid biocomposite exhibited antibacterial activities, which assisted in bacterial cell death.

### 3.4. MMT Assay (Cell Availability)

**Figure 7:**
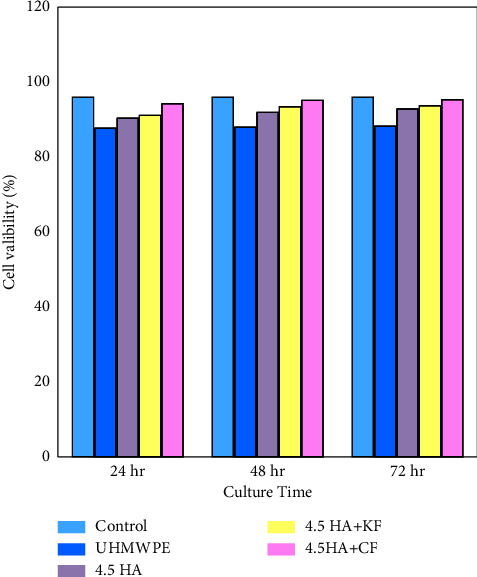
Cell availability of biocomposite bone plates fixation as a function of hydroxyapatite nanoparticles.

**Figure 8:**
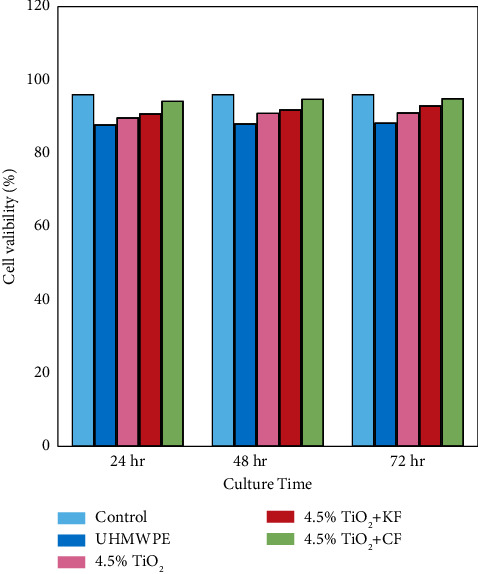
Cell availability of biocomposite bone plates fixation as a function of titanium dioxide nanoparticles.

Biocompatibility was evaluated by assessing the cell viability that can be defined as a time-dependent phenomenon. Figures 7 and 8 show the cell viability of all biocomposites, and results revealed that pure UHMWPE exhibited 91.37%, 91.65%, and 91.92 viabilities at 24, 48, and 72 h, respectively. When HA and TiO_2_ nanoparticles were added, the results showed a considerable increase in cell availability, which increased as the number of days increased [22]. In addition, the viability results showed higher cell viability when particulate biocomposite reinforced with carbon fiber than with Kevlar fiber for both types of nanoparticle samples at 24, 48, and 72 hr. [31]. Furthermore, 4.5% n-HA + CF hybrid biocomposites showed excellent cell availability (99.29%) at 72 hr. Also, none of the specimens show any significant toxicity toward human fibroblast cells. The result can be explained by hybrid biocomposites maintaining superior aviability after culturing, making them very good candidates for bone plate fixation in vivo.

## 4. Conclusion

This study aimed at the development of UHMEPE nanobiocompsite materials which firstly used as internal bone plate fixation. According to the obtained data, it was found that

- The study of the antibacterial activity against *E. coli* and *S. aureus* bacteria recorded an improvement in bone plates biocomposite compared with pure UHMWPE, and the best result obtained with hybrid biocomposites bone plate fixation containing multiple reinforcement titanium dioxide (n-TiO_2_) and carbon fiber.
- Contact angle decreased with an increase in the nanoparticles content, which led to an increase in hydrophobicity. Whereas hybrid biocomposites for bone plate fixation enhance wettability, a hybrid biocomposite (UHMWPE/CF) with n-TiO_2_ would be more hydrophilic and therefore have high surface energy.
- The in vitro investigation with MTT assay reveals a high cytocompatibility of the prepared biocomposite specimens whereas the incorporation of n-HA and n-TiO_2_ in UHMWPE matrix became more active after 72 hr. of exposure in human fibroblast cells, and there was a remarkable increase in cell viability when hybrid biocomposites bone plate fixation containing multiple reinforcement hydroxyapatite (n-HA) and carbon fiber. It seems that a material composition enhances cells' growth and activity without any toxic effects on the cells.
- DSC analysis results revealed an improvement in the thermal stability of bionanocomposites, and the melting temperature *T*_*m*_ and crystallization temperature *T*_*c*_ have been enhanced by the addition of n-HA and carbon fiber.

## Figures and Tables

**Table 1:** DSC results of particulates biocomposite and hybrid biocomposites.

Specimens	*T*_*m*_	*T*_*C*_
UHMWPE	136.29	235
UHMWPE + 1.5 HA	137	237
UHMWPE + 2.5 HA	140	239
UHMWPE + 3.5 HA	143	237
UHMWPE + 4.5 HA	145	239
UHMWPE + 4.5 HA + KF	147	240
UHMWPE + 4.5 HA + CF	148.27	245
UHMWPE + 1.5 TiO_2_	137	237
UHMWPE + 2.5 TiO_2_	142	238
UHMWPE + 3.5 TiO_2_	144	237
UHMWPE + 4.5 TiO_2_	144.82	239
UHMWPE + 4.5 TiO_2_ + KF	146	237
UHMWPE + 4.5 TiO_2_ + CF	147	240

## Data Availability

The data that support the findings of this study can be obtained from the corresponding author upon request.

## References

[1] Kim S. H., Chang S. H., Jung H. J. (2010). The finite element analysis of a fractured tibia applied by composite bone plates considering contact conditions and time-varying properties of curing tissues. *Composite Structures*.

[2] Qiao B., Zhou D., Dai Z. (2019). Bone Plate composed of a ternary nanohydroxyapatite/polyamide 66/glass fiber composite: biocompatibility in vivo and internal fixation for canine femur fractures. *Advanced Functional Materials*.

[3] Hashim A. M., Tanner E. K., Oleiwi J. K. (2016). Biomechanics of natural fiber green composites as internal bone plate rafted. *MATEC Web of Conferences*.

[4] Haas N., Hauke C., Schütz M., Kääb M., Perren S. M. (2001). Treatment of diaphyseal fractures of the forearm using the Point Contact Fixator (PC-Fix): results of 387 fractures of a prospective multicentric study (PC-Fix II). *Injury*.

[5] Bonfield W., Grynpas M. D., Tully A. E., Bowman J., Abram J. (1981). Hydroxyapatite reinforced polyethylene--a mechanically compatible implant material for bone replacement. *Biomaterials*.

[6] Kabiri A., Liaghat G., Alavi F. (2020). Glass fiber/polypropylene composites with potential of bone fracture fixation plates: manufacturing process and mechanical characterization. *Journal of Composite Materials*.

[7] Kadhim Oleiwi J., Alwan M. K., Adnan Hamad Q. (2014). Numerically and experimentally studying of some mechanical properties of the polyester matrix composite material reinforced by jute fibers. *Journal*.

[8] Liu J. l., Zhu Y. y., Wang Q. l., Ge S. r. (2008). Biotribological behavior of ultra high molecular weight polyethylene composites containing bovine bone hydroxyapatite. *Journal of China University of Mining and Technology*.

[9] Chang B. P., Md Akil H., Md Nasir R. B. (2013). Comparative study of micro- and nano-ZnO reinforced UHMWPE composites under dry sliding wear. *Wear*.

[10] Jawad Kadhim Oleiwi et al J. K. O. e. a., Anaee R. A., Radhi S. H. (2018). Roughness, wear and thermal analysis of uhmwpe nanocomposites asacetabular cup in HIP joint replacement. *International Journal of Mechanical and Production Engineering Research and Development*.

[11] Kim H. W., Kim H. E., Salih V. (2005). Stimulation of osteoblast responses to biomimetic nanocomposites of gelatin-hydroxyapatite for tissue engineering scaffolds. *Biomaterials*.

[12] Celebi Efe G., Altinsoy I., Türk S., Bindal C., Ucisik A. H. (2019). Effect of particle size on microstructural and mechanical properties of UHMWPE–TiO2 composites produced by gelation and crystallization method. *Journal of Applied Polymer Science*.

[13] Bahjat H. H., Ismail R. A., Sulaiman G. M., Jabir M. S. (2021). Magnetic field-assisted laser ablation of titanium dioxide nanoparticles in water for anti-bacterial applications. *Journal of Inorganic and Organometallic Polymers and Materials*.

[14] Khashan K. S., Abdulameer F. A., Jabir M. S., Hadi A. A., Sulaiman G. M. (2020). Anticancer activity and toxicity of carbon nanoparticles produced by pulsed laser ablation of graphite in water. *Advances in Natural Sciences: Nanoscience and Nanotechnology*.

[15] Jihad M. A., Noori F. T. M., Jabir M. S., Albukhaty S., AlMalki F. A., Alyamani A. A. (2021). Polyethylene glycol functionalized graphene oxide nanoparticles loaded with nigella sativa extract: a smart antibacterial therapeutic drug delivery system. *Molecules*.

[16] Mannoush S. H., Thaker A. A., Jabir M. S. (2022). Inhibition of ovarian cancer cells growth using gold nanoparticles and silica coated gold nanoparticles: in-vitro study. *Journal of Pharmaceutical Negative Results*.

[17] Huhtamäki T., Tian X., Korhonen J. T., Ras R. H. A. (2018). Surface-wetting characterization using contact-angle measurements. *Nature Protocols*.

[18] Astm D. (2017). Standard test method for corona-treated polymer films using water contact angle measurements. https://webstore.ansi.org/standards/astm/astmd594604.

[19] Hasanzadeh A., Radmanesh F., Kiani J. (2019). Photoluminescent functionalized carbon dots for CRISPR delivery: synthesis, optimization and cellular investigation. *Nanotechnology*.

[20] Astm E. (2003). *Standard Test Method for Assignment of the Glass Transition Temperature by Differential Scanning Calorimetry*.

[21] Al-Mutairi N. H., Najim M., Sabr O. H., Kareem F. A. (2022). Mechanical properties and wear behavior of polypropylene/hydroxyapatite nanocomposite. *Egyptian Journal of Chemistry*.

[22] Ortiz-Hernández R., Ulloa-Castillo N. A., Diabb-Zavala J. M. (2019). Advances in the processing of UHMWPE-TiO2 to manufacture medical prostheses via SPIF. *Polymers*.

[23] Kang X., Zhang W., Yang C. (2016). Mechanical properties study of micro- and nano-hydroxyapatite reinforced ultrahigh molecular weight polyethylene composites. *Journal of Applied Polymer Science*.

[24] Aisyah H. A., Paridah M. T., Sapuan S. M. (2019). Thermal properties of woven kenaf/carbon fibre-reinforced epoxy hybrid composite panels. *International Journal of Polymer Science*.

[25] Cano L., Pollet E., Avérous L., Tercjak A. (2017). Effect of TiO2 nanoparticles on the properties of thermoplastic chitosan-basednano-biocomposites obtained by mechanical kneading. *Composites Part A: Applied Science and Manufacturing*.

[26] Ma R., Guo D. (2019). Evaluating the bioactivity of a hydroxyapatite-incorporated polyetheretherketone biocomposite. *Journal of Orthopaedic Surgery and Research*.

[27] Prodana M., Duta M., Ionita D. (2015). A new complex ceramic coating with carbon nanotubes, hydroxyapatite and TiO2 nanotubes on Ti surface for biomedical applications. *Ceramics International*.

[28] de Dicastillo C. L., Correa M. G., Martínez F. B., Streitt C., Galotto M. J. (2020). Antimicrobial effect of titanium dioxide nanoparticles. *Antimicrobial Resistance - A One Health Perspective*.

[29] Mohammed A. A., Oleiwi J. K., Al-Hassani E. S. (2021). The effect of nanoparticles (n-HAp, n-TiO_2_) on the thermal properties and biomechanical analysis of polymeric composite materials for dental applications. *Nano Hybrids Compos*.

[30] Salih S., Oleiwi J., Ali H. (2019). Development the physical properties of polymeric blend (SR/PMMA) by adding various types of nanoparticles, used for maxillofacial prosthesis applications. *Engineering and Technology Journal*.

[31] Cecen B., Kozaci D., Yuksel M., Erdemli D., Bagriyanik A., Havitcioglu H. (2015). Biocompatibility of MG-63 cells on collagen, poly-L-lactic acid, hydroxyapatite scaffolds with different parameters. *Journal of Applied Biomaterials & Functional Materials*.

